# Successful polyethylene glycol fusion repair using stored viable peripheral nerve allografts in Sprague–Dawley and Lewis rats

**DOI:** 10.4103/NRR.NRR-D-24-01505

**Published:** 2025-07-05

**Authors:** Liwen Zhou, Cathy Z. Yang, Alexander M. Schafer, Alexa N. Olivarez, Arjun Agarwal, Guhan Periyasamy, Karthik Venkudusamy, Yessenia Montoya, Varun Gokhale, Rhea Sood, Henry Garcia, Jared S. Bushman, George D. Bittner

**Affiliations:** 1 Department of Neuroscience, The University of Texas at Austin, Austin, TX, USA; 2 School of Pharmacy, University of Wyoming, Laramie, WY, USA

**Keywords:** axonal morphometrics, axotomy, Lewis, peripheral nerve injury, peripheral nerve repair, polyethylene glycol fusion, sciatic nerve ablation, Sprague–Dawley, tissue storage solutions

## Abstract

We have previously shown the success of polyethylene glycol fusion repair of segmental-loss peripheral nerve injuries in rats using freshly harvested, viable peripheral nerve allografts that can conduct action potentials. Because clinical application of polyethylene glycol fusion with viable peripheral nerve allografts demands pre-transplant donor tissue storage, we developed a protocol for *ex vivo* storage of rat sciatic nerves as viable peripheral nerve allografts, preserving many axons for up to 5 days. The current study evaluated the *in vivo* use of these stored viable peripheral nerve allografts. We hypothesized that stored viable peripheral nerve allografts with viable axons would enable successful *in vivo* repair of segmental-loss peripheral nerve injuries via polyethylene glycol-fusion. Polyethylene glycol-fused viable peripheral nerve allografts were classified as successful if they produced significantly improved locomotor recovery, as evaluated by the sciatic functional index, within 8 weeks post-repair. Many Sprague–Dawley and Lewis rats with successfully polyethylene glycol-fused viable peripheral nerve allografts had significantly improved sciatic functional index scores beginning at 5 weeks post-operatively. There was no significant difference in the efficiency and extent of successful polyethylene glycol fusion between stored and freshly harvested viable peripheral nerve allografts. In contrast, rats with non-fused negative control viable peripheral nerve allografts showed no recovery by 8 weeks post-operatively. Additional confirmatory outcome measures included *in vivo* compound action potentials and assessments of axon morphometry. These results suggest that viable peripheral nerve allografts can be stored and later used for successful polyethylene glycol fusion repair of segmental-loss peripheral nerve injuries.

## Graphical abstract

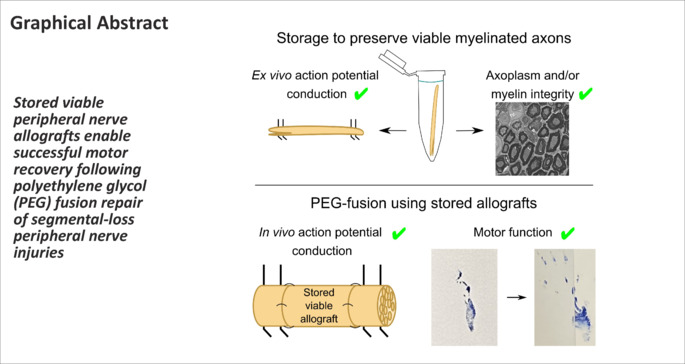

## Introduction

Segmental-loss peripheral nerve injuries (SL-PNIs) often result in nerve defects that require bridging materials to restore lost motor and sensory behaviors (Campbell, 2008; Taylor et al., 2008; Birch et al., 2012a, b; Muheremu and Ao, 2015; Jones et al., 2018; Wang et al., 2019; Pan et al., 2020). Current strategies focus on enhancing axonal regeneration (Godinho et al., 2013; Rao et al., 2022; Su et al., 2022; Garcia-Garcia et al., 2023; Perrelle et al., 2023), but rarely achieve significant behavioral recovery partly because regenerating axons often fail to reach distal targets before they atrophy (Sakuma et al., 2016; Mikesh et al., 2018; Allgood et al., 2022). Nerve autografts are the current gold standard to repair SL-PNIs, but they have limitations, including appropriate size-matching, expense, and donor site morbidity. Viable peripheral nerve allografts (VPNAs) provide a more readily available and size-matched alternative, but they are not typically used experimentally or clinically due to immune rejection and comorbidity from immunosuppression (Duncan and Wilkes, 2005; Brushart, 2011; Bittner et al., 2022).

We have developed a technology (PEG-fusion of VPNAs) to repair SL-PNIs that applies a series of bio-engineered solutions, one being polyethylene glycol (PEG), during surgery. This approach, combined with neurorrhaphy, non-selectively fuses/repairs donor and host axons at the proximal and distal ends of a VPNA. The ability of VPNAs to conduct compound action potentials (CAPs) is crucial for PEG-fusion to achieve immediate restoration of axolemmal and electrophysiological continuity across the SL-PNI (Bittner et al., 2016, 2018). PEG-fusion repair differs from conventional repairs (neurorrhaphy only without axon fusion) that assume all donor graft and host distal axons undergo rapid Wallerian degeneration (WD). PEG-fusion repair of SL-PNIs produces 1) immediate reinnervation of denervated distal motor targets, 2) rescue of PEG-fused axons from WD, 3) faster and improved motor recovery compared to non-fused negative controls (NCs), and 4) immunotolerance of PEG-fused VPNAs without immunosuppression or tissue matching (Ghergherehchi et al., 2016, 2019; Mikesh et al., 2018; Smith et al., 2020a, b).

We have previously reported that axonal viability in VPNAs from Sprague–Dawley rats can be maintained up to 9 days *ex vivo*, as assessed by CAPs and axonal morphology, when stored in calcium-free, hypotonic, diluted Normosol-R at 4°C (Zhou et al., 2023). Many axons in VPNAs were well-preserved for up to 5 days. No previous study has examined whether stored VPNAs can be successfully PEG-fused. In the present study, we used two immunologically-mismatched strains of rats as models for VPNA transplantation: Sprague–Dawley donor to Sprague–Dawley host (SD-d/SD-h) and Sprague–Dawley donor to Lewis host (SD-d/Lewis-h).

We hypothesized that stored VPNAs with viable axons would enable successful *in vivo* PEG-fusion repair of SL-PNIs, resulting in behavioral motor recovery. Our data showed that successful motor recovery, as assessed by the sciatic functional index (SFI), can be achieved with PEG-fusion repair using stored VPNAs in both rat strains. Longer VPNA storage times did not significantly alter the rate or timing of successful SFI recovery. Many large-diameter donor grafts and host distal axons were rescued from WD after successful PEG-fusion but not NC repairs. VPNA storage times did not significantly alter average axon diameter, density, or the percentage of PEG-fused axons. Our findings are consistent with our hypothesis and suggest that PEG-fusion VPNA repair could improve clinical outcomes for SL-PNIs.

## Methods

### Animals

All experimental procedures adhered to the National Institutes of Health Guide for the Care and Use of Laboratory Animals (8^th^ ed., National Research Council, 2011) and were approved by the Institutional Animal Care and Use Committee (IACUC) at the University of Texas at Austin (AUP-2022-00278, approved February 7, 2023). Male (250–500 g) and female (225–300 g) outbred SD and inbred Lewis rats aged 3–12 months were housed 2–3/cage and maintained on a 12-hour light/dark cycle with food and water given *ad libitum*. A total of 197 rats (123 SD and 74 Lewis) were used in this study. Surgical and behavioral procedures were performed during the day.

Only outbred SD rats were used as donors for VPNAs, while both outbred SD and inbred Lewis rats were used as hosts for donor VPNAs. Operated rats were accordingly denoted as SD-d/SD-h (SD donor to SD host) or SD-d/Lewis-h (SD donor to Lewis host). The number of SD-d/SD-h rats decreased with PO time due to self-mutilation to hind paw digits that required premature euthanasia in accordance with IACUC guidelines. Consequently, most surgeries performed were SD-d/Lewis-h rats that did not exhibit self-mutilation behaviors.

### Nerve extraction and storage

Sciatic nerve segments (VPNAs, *n* = 141) were harvested from SD rats as previously described (Zhou et al., 2023). Because we found no difference in CAP conduction between VPNAs harvested before or after euthanasia, all VPNAs were harvested after euthanasia. Euthanasia was performed under deep anesthesia with a 4% isoflurane (RXISO-250; Animal Health International, Roanoke, TX, USA)/oxygen mixture at 1.5 L/min (Handlebar Anesthesia, Pflugerville, TX, USA) followed by an intracardiac KCl injection. Prior to VPNA harvesting, all surgical tools were autoclaved. The incision site was shaved and sterilized by several wipes of 70% ethanol followed by Betadine. A 4–5 cm incision was made through the biceps femoris to expose the sciatic nerve. Left and right sciatic nerve segments 1.8–2.5 cm in length were excised and stored in Eppendorf tubes at 4°C in 2 mL of diluted Normosol-R (250–255 mOsm) containing 1x final concentration of penicillin-streptomycin (P4333; Sigma-Aldrich, St. Louis, MO, USA; see Zhou et al. (2023) for more solution detail). Fresh diluted Normosol-R solution was prepared and replaced daily.

### Electrophysiological recordings

*Ex vivo* CAPs were recorded daily as previously described (Zhou et al., 2023). Briefly, VPNAs were placed in a custom 3D-printed chamber (ABS polymer; Craftbot XL machine; Budapest, Hungary) with titanium wires connected to a set of stimulating and recording electrodes at two ends of the nerve. VPNAs were stimulated with incremental voltages from 0–8 V using 0.1 ms square wave depolarizations given at 1 Hz with a 0.1 ms delay from a sweep-triggering pulse using a PowerLab 4/35 (ADInstruments, Sydney, Australia) and recorded using a Dual Bio Amp (ADInstruments). To avoid nerve damage, stimulus amplitudes were limited to 1 V maximum on day 0 and 8 V maximum at later storage times. VPNAs that failed to conduct CAPs immediately after harvest on day 0 were excluded from further analyses.

*In vivo* CAPs were recorded as previously described (Mikesh et al., 2018; Ghergherehchi et al., 2019; Hibbard et al., 2025; Zhou et al., 2025). Briefly, *in vivo* CAPs were recorded before sciatic ablation and immediately following PEG-fusion to confirm electrophysiological continuity. Stimulating and recording electrodes were placed proximally and distally to the graft at a distance of ≥ 1 cm apart. Sciatic nerves were stimulated at 1 V using 0.1 ms square wave depolarizations at 1 Hz with a 0.1 ms delay from the trigger signal using a PowerLab 4/35 and recorded using a Dual Bio Amp.

### Surgical procedure

Surgical procedures were performed as previously described (Mikesh et al., 2018; Ghergherehchi et al., 2019; Hibbard et al., 2025; Smith et al., 2025). Briefly, SD-h and Lewis-h rats were anesthetized with isoflurane (4% induction, 2% maintenance)/oxygen mixture at 1.5 L/min. The lateral aspect of the left hindlimb was shaved and sterilized, and a 2–3 cm incision was made through the skin and the biceps femoris to expose the sciatic nerve. The sciatic nerve was sharply ablated (~4 mm) at the mid-thigh level by fine dissection scissors in a Ca^2+^-containing isotonic extracellular solution (0.9% NaCl with 2 mM CaCl_2_). In both PEG-fused and NC groups, severed sciatic nerve stumps were irrigated with 0.5% methylene blue followed by diluted Normosol-R. Size- and sex-matching SD-d VPNAs stored for up to 5 days were trimmed to an appropriate length for transplantation. To minimize tension on host and donor axons after neurorrhaphy, SD-d VPNAs were made 1–3 mm longer than the ablated gap. Both ends of the host sciatic nerve stumps were trimmed flush to enable their close apposition to SD-d VPNAs with at least four 10-0 microsutures through the epineurium and/or perineurium sheaths. For PEG-fused rats, lesion sites were then submerged in a sterile solution of 50% w/w 3.35 kDa PEG (Sigma-Aldrich) in distilled water for 1–2 minutes to non-specifically fuse the closely apposed, open axonal ends. NC rats underwent neurorrhaphy but were not treated with PEG. Following neurorrhaphy, lesion sites in both PEG-fused and NC rats were flushed several times with a Ca^2+^-containing isotonic extracellular solution to accelerate Ca^2+^-induced vesicle accumulation and seal any remaining open axons (Mencel and Bittner, 2023). The muscle incision was closed with 5-0 sutures, and the skin was closed with wound clips. Rats recovered from surgery on heat pads before being returned to standard housing. Rats received 5 mg/kg subcutaneous injections of carprofen during surgery and daily for the next 3 days.

### Morphological analyses

Nerve samples were fixed and embedded as previously described (Mikesh et al., 2018; Smith et al., 2020a, 2025; Zhou et al., 2023, 2025). Briefly, samples were fixed in 2% paraformaldehyde/3% glutaraldehyde in 0.1 M sodium cacodylate buffer. Portions of stored VPNAs not used for surgical procedures were trimmed into 5 mm segments. Sciatic nerves from rats harvested at 8 weeks PO were divided to obtain 4–5 mm proximal, graft, and distal segments each 1–2 mm away from the suture sites. All samples were post-fixed in 1% osmium tetroxide/1% potassium ferrocyanide followed by 1% aqueous uranyl acetate prior to dehydration and embedding in Hard Plus Resin 812 (Electron Microscopy Sciences, Hatfield, PA, USA). Samples were incubated at 60°C for 48–72 hours prior to sectioning. Glass knife thick sections (0.5 μm) were obtained using a Leica ultramicrotome, stained with toluidine blue, and imaged on a Zeiss Axiovert 200M fluorescent light microscope with an HR3 camera (Hebron, KY, USA). Axonal morphology was analyzed using ImageJ (version 1.53m, National Institutes of Health, Bethesda, MD, USA) on randomly chosen regions of interest (ROIs).

*Ex vivo* stored VPNAs were analyzed based on five axon categories as percentages of the total axon count (Zhou et al., 2023). Three ROIs per sample containing 150–200 axons were analyzed in three samples per time point. For sciatic nerve samples collected at 8 weeks PO, axon diameter, axon density, and percentage of large-diameter axons were assessed across treatment groups (Mikesh et al., 2018; Smith et al., 2020a, 2025; Zhou et al., 2025). For axon diameter, 150–200 axons from at least 3 ROIs per sample were analyzed in two samples per treatment group. For axon density, five ROIs per sample containing 300–550 axons were analyzed in two samples per treatment group. For large-diameter axons, at least five ROIs per sample containing at least 250 axons were analyzed in 2 samples per treatment group.

### Behavioral analyses

The SFI was performed as previously described (Hibbard et al., 2025; Smith et al., 2025; Zhou et al., 2025). Rats were handled and trained for at least three sessions prior to surgery to become acclimated to the testing apparatus and procedure. SFI tests were performed and scored by testers blinded to the experimental groups. Rat hind paws were marked with red ink (right, unoperated side) and blue ink (left, operated side). Rats were placed on one end of a slightly inclined board (1.52 m long, 10.2 cm wide) lined with paper strips and allowed to run back to the home cage on the other end. Inked paw prints were analyzed for paw length, total toe spread, and intermediate toe spread to compute SFI scores. A successful SFI trial required three consecutive steps by each hindlimb without hesitating or stopping. Two trials were performed for each rat at each time point, and scores were averaged to obtain the final score. Unoperated rats exhibit SFI scores of 0 ± 30, indicating symmetry of gait. Impaired movement of the injured hind paw results in lower, negative SFI scores. Rats were tested weekly after surgery for up to 8 weeks PO.

### Statistical analyses

All statistical analyses were performed using GraphPad Prism (version 8, GraphPad Software, Boston, MA, USA, www.graphpad.com). Data from male and female rats were pooled for both SFI and morphological analyses, as previous studies reported no related sex differences (Hibbard et al., 2025; Smith et al., 2025; Zhou et al., 2025). *Ex vivo* VPNA survival curves were compared using the log-rank test and the Gehan-Breslow-Wilcoxon test. SFI data were analyzed by mixed-effects analysis for SD-d/SD-h rats and two-way analysis of variance with repeated measures in SD-d/Lewis-h rats followed by *post hoc* Tukey’s multiple comparisons. This difference in analysis methods was necessary due to missing data points in SD-d/SD-h rats caused by self-mutilation, which required early euthanasia. The success rates of PEG-fusion surgeries were analyzed by chi-square test. Morphological data were analyzed by one-way analysis of variance followed by *post hoc* Tukey’s multiple comparisons. A 95% confidence interval was used for all analyses. All data are presented as mean ± SEM. The number of animals and/or axons analyzed are indicated in each figure panel or legend.

## Results

### Assessments of axonal viability for viable peripheral nerve allografts stored *ex vivo*

#### Electrophysiological assessments

**Figure 1:**
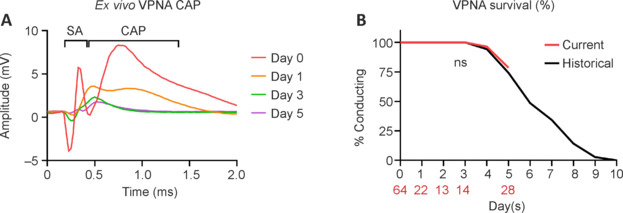
Electrophysiological confirmation of VPNA survival *ex vivo*. (A) Representative CAP recordings *ex vivo* from Sprague–Dawley donor VPNAs stored for 0 to 5 days in 4°C diluted Normosol-R. (B) Comparison of current and historical data by Zhou et al. (2023) for percentage of VPNAs that conducted *ex vivo* CAP for up to 9 days. ns: Not significant according to the log-rank test and the Gehan-Breslow-Wilcoxon test. *n* numbers of harvested VPNAs in the current study are indicated below each time point in red font. CAP: Compound action potential; SA: stimulus artifact; VPNA: viable peripheral nerve allograft.

SD donor VPNAs stored for 0 to 5 days in 4°C diluted Normosol-R were assessed daily for *ex vivo* CAP conduction prior to transplantation (**Figure 1A**). While CAPs were maintained for that time, CAP peaks for longer storage times required higher stimulating voltages (up to 8 V). Thus, *ex vivo* CAPs were assessed qualitatively. CAP peaks for longer stored VPNAs displayed a trend of reduced amplitudes. 100% of VPNAs stored for 0 to 3 days and 79% of VPNAs stored for 5 days conducted *ex vivo* CAPs. These results were consistent with our historical results (**Figure 1B**) in which 100% of VPNAs stored for 0 to 3 days and 74% of VPNAs stored for 5 days conducted *ex vivo* CAPs (Zhou et al., 2023). We did not store VPNAs beyond 5 days in this study because the percentage of VPNAs that maintained CAP conduction decreased rapidly after 5 days in our historical assessments using the same storage protocol (**Figure 1B**).

#### Morphological assessments

**Figure 2:**
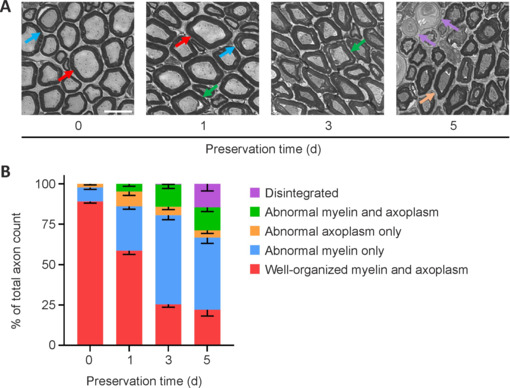
Morphology of VPNAs stored *ex vivo*. (A) Representative cross sections (0.5 µm) of Sprague–Dawley donor VPNAs stored for 0 to 5 days in 4°C diluted Normosol-R. Scale bar: 10 μm. Red arrows point to axons with well-organized myelin and axoplasm, blue arrows point to axons with abnormal myelin, orange arrow points to axon with abnormal axoplasm, green arrows point to axons with abnormal myelin and axoplasm, and purple arrows point to disintegrated axons. (B) Quantification of axons according to different morphological categories assessing myelin and axoplasm. Two-way analysis of variance interaction *F*_(12,160)_ = 78, *P* < 0.0001; time *F*_(3, 160)_ = 0, ns: Not significant; category *F*_(4,160)_ = 338, *P* < 0.0001. Values are mean ± SEM. *n* of three regions of interest (quantifying at least 150 axons in total) per sample in 3 samples per time point. VPNA: Viable peripheral nerve allograft.

SD donor VPNAs stored for 0 to 5 days were sampled for morphological analyses based on our previously established five-category classification (Zhou et al., 2023) that consists of 1. axons with well-organized myelin, 2. axons with abnormal myelin, 3. axons with abnormal axoplasm, 4. axons with abnormal myelin and axoplasm, and 5. axons with disintegrated myelin (**Figure 2**). “Axons with disintegrated myelin” was redefined for this current paper as “Disintegrated axons” because both myelin and axoplasm had severely degraded. Samples were taken from unused segments of VPNAs prepared for surgeries. VPNAs stored for up to 5 days appeared similar to those in the previous study (**Figure 2A**; Zhou et al., 2023). Percentages of axons in each morphological category were quantified (**Figure 2B**), and pair-wise statistical comparisons were performed (**Table 1**). The percentage of well-organized axons decreased significantly (*P* < 0.0001) with longer storage times, starting at 89% ± 1.1% in fresh (0-day) VPNAs and declining to 22% ± 3.9% in 5-day VPNAs. Axons with abnormal myelin were evident in 0-day VPNAs (8.7% ± 1.2%) and were significantly (*P* < 0.01 or better) more prominent in VPNAs having longer storage times (> 40% in 3-day and 5-day VPNAs). Axons with abnormal axoplasm were less prevalent in 0-day VPNAs (2.2 ± 0.6%) and did not increase significantly (*P* > 0.05) with longer storage times (4%–5% in 3-day and 5-day VPNAs). Axons with both abnormal myelin and axoplasm were absent in 0-day VPNAs but became significantly (*P* < 0.001 or *P* < 0.0001) present in 3-day VPNAs (14% ± 2.4%) and 5-day VPNAs (14% ± 2.6%). Disintegrated axons were only present in 5-day VPNAs (15% ± 4.3%). These findings aligned with previous results and confirmed the axonal viability of VPNAs stored in 4°C diluted Normosol-R (Zhou et al., 2023).

**Table 1:** Pair-wise statistical comparisons summarizing stored VPNA morphology

Comparison			Well-organized	Abnormal myelin	Abnormal axoplasm	Abnormal myelin + axoplasm	Disintegrating axons
Day 0	*vs.*	Day 1	****	****	ns	ns	ns
		Day 3	****	****	ns	***	ns
		Day 5	****	****	ns	****	****
Day 1	*vs.*	Day 3	****	****	ns	*	ns
		Day 5	****	****	ns	*	****
Day 3	*vs.*	Day 5	ns	**	ns	ns	****

Two-way analysis of variance followed by Tukey’s multiple comparison test. *P < 0.05, **P < 0.01, ***P < 0.001, ****P < 0.0001. ns: Not significant; VPNA: viable peripheral nerve allograft.

### Assessments of axonal viability for stored viable peripheral nerve allografts polyethylene glycol-fused *in vivo*

#### Behavioral assessments

**Figure 3:**
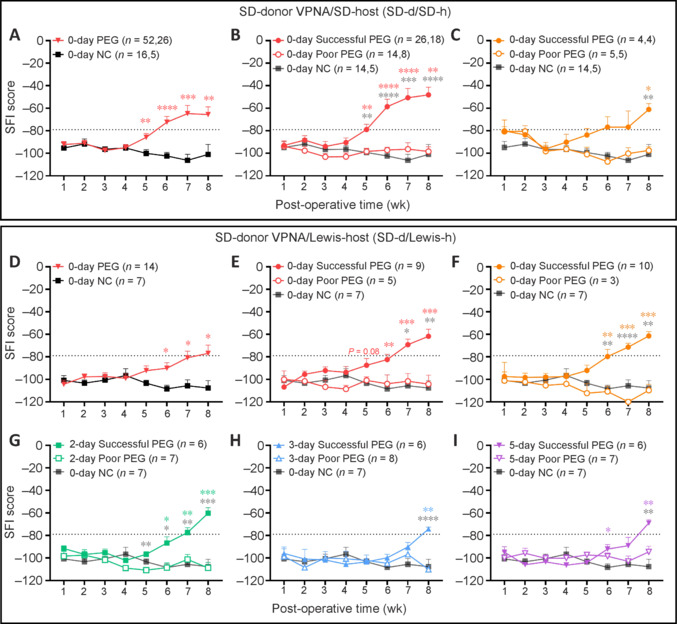
Repairs using stored VPNAs that are PEG-fused successfully restore locomotor behavior as assessed by SFI. SD host (A–C) and Lewis host (D–I) rats received PEG-fused or non-fused NC VPNA repairs immediately following SL-PNIs. 0-day NC rats of the same host strains were plotted accordingly across panels for statistical comparisons. All panels included a dashed line at an SFI score of –79 to indicate the criteria for successful PEG-fusion. (A) 0-day PEG-fused rats *vs.* 0-day NC rats. The numbers of rats generated for 6 and 8 weeks PO endpoints are listed. This includes rats euthanized early due to self-mutilation. Mixed-effects interaction *F*_(7,234)_ = 10.3, *P* < 0.0001; time *F*_(2.7, 92)_ = 4.5, *P* = 0.0069; treatment *F*_(1,40)_ = 23, *P* < 0.0001. 0-day (B) and 1-day (C) PEG-fused rats were divided into successful PEG and poor PEG groups. Only the number of rats that reached 6 and 8 weeks PO endpoints are listed and analyzed. Mixed-effects analysis. For B, interaction *F*_(14,306)_ = 7.9, *P* < 0.0001; time *F*_(3.1, 133)_ = 4.5, *P* = 0.0078; treatment *F*_(2,51)_ = 19, *P* < 0.0001. For C, interaction *F*_(14,121)_ = 2.1, *P* = 0.0169; time *F*_(3.7, 64)_ = 2.5, ns; treatment *F*_(2,20)_ = 7.2, *P* = 0.0043. (D) 0-day PEG-fused rats *vs.* 0-day NC rats. Two-way analysis of variance interaction *F*_(7,133)_ = 4.5, *P* = 0.0002; time *F*_(2.9, 54)_ = 1.4, ns; treatment *F*_(1,19)_ = 5.0, *P* = 0.0378. (E–I) 0-day to 5-day PEG-fused rats were divided into successful PEG and poor PEG groups. Two-way analysis of variance. For E, interaction *F*_(14,126)_ = 5.5, *P* < 0.0001; time *F*_(3.8, 68)_ = 2.3, ns; treatment *F*_(2,18)_ = 12, *P* = 0.0006. For F, interaction *F*_(14,119)_ = 5.7, *P* < 0.0001; time *F*_(4.3, 73)_ = 0.9, ns; treatment *F*_(2,17)_ = 21, *P* < 0.0001. For G, interaction *F*_(14,117)_ = 6.0, *P* < 0.0001; time *F*_(4.8, 80)_ = 3.2, *P* = 0.0122; treatment *F*_(2,17)_ = 18, *P* < 0.0001. For H, interaction *F*_(14,126)_ = 3.0, *P* = 0.0006; time *F*_(4.4, 80)_ = 1.3, ns; treatment *F*_(2,18)_ = 1.9, ns. For I, interaction *F*_(14,119)_ = 4.4, *P* < 0.0001; time *F*_(3.8, 64)_ = 2.7, *P* = 0.0404; treatment *F*_(2,17)_ = 3.3, ns. For D–I, n numbers are listed for rats that reached endpoints at 8 weeks PO. Values are mean ± SEM. * at any time point indicate P values between successful PEG and 0-day NC (colored) and between successful PEG and poor PEG (gray). Tukey’s multiple comparison test. *P < 0.05, **P < 0.01, ***P < 0.001, ****P < 0.0001. NC: Negative control; PEG: polyethylene glycol; PO: post-operatively; SD: Sprague–Dawley; SFI: sciatic functional index; VPNA: viable peripheral nerve allograft.

Both SD and Lewis rats were used as hosts (SD-h and Lewis-h) to receive SD VPNAs (SD-d) that conducted *ex vivo* CAP. For PEG-fusion repairs of SL-PNIs, fresh (0-day) and stored (up to 5 days) SD-d VPNAs were transplanted. Only fresh (0-day) SD-d VPNAs were transplanted in non-fused NC VPNA repairs because NC repairs do not rescue viable axons in donor VPNAs from rapid WD. Because recovery following NC repairs solely relies on axonal regeneration by outgrowth, longer storage of VPNAs for NC repairs was not expected to improve SFI within the 8-week threshold set for this study. **Figure 3** shows SFI locomotor recovery in SD-d/SD-h (**Figure 3A–C**) and SD-d/Lewis-h (**Figure 3D–I**) rats using VPNAs stored for up to 5 days. Group names describe storage time and the method of repair (e.g., “0-day PEG” indicates PEG-fused VPNAs stored for 0 days). Historical data from previous studies (Mikesh et al., 2018; Hibbard et al., 2025) were included for 0-day PEG-fused (*n* = 13) and 0-day NC (*n* = 12) SD-d/SD-h rats (**Figure 3A** and **B**); all other animals were generated in this study.

We began this study using SD-d/SD-h allograft protocols as previously published (Riley et al., 2015; Mikesh et al., 2018; Smith et al., 2020a, 2025; Hibbard et al., 2025). However, 21% (14/67) of SD-d/SD-h rats exhibited severe self-mutilation that resulted in early euthanasia. In contrast, no self-mutilation was observed in SD-d/Lewis-h rats (0/74; 0%), consistent with previous findings (Panerai et al., 1987; Carr et al., 1992; Zhou et al., 2025). To minimize animal discomfort and early sacrifice, the study was continued with SD-d/Lewis-h rats only. Both SD-d/SD-h and SD-d/Lewis-h transplants have genetic mismatches and are allografts (Roballo and Bushman, 2019).

In SD-d/SD-h rats, 0-day PEG-fused and 0-day NC rats exhibited similar SFI scores between 1–4 weeks PO (**Figure 3A**). 0-day PEG-fused rats exhibited significantly (*P* < 0.01 or better) higher SFI scores than 0-day NC rats starting at 5 weeks PO. At 8 weeks PO, 0-day PEG-fused and 0-day NC rats scored –66 ± 6.9 and –101 ± 8.8, respectively. Consistent with previous observations (Mikesh et al., 2018; Smith et al., 2025; Zhou et al., 2025), PEG-fused rats exhibited two types of distinct SFI recovery trajectories. Using previously established criteria, we divided all PEG-fused rats into Successful PEG-fused (reached and maintained SFI scores above -79) and Poor PEG-fused groups (failed to reach SFI scores above -79) (Mikesh et al., 2018; Smith et al., 2025). For context, non-fused NC rats almost never reach this SFI threshold of more than -79. 0-day Successful PEG-fused rats exhibited significantly higher (*P* < 0.01 or better) SFI scores than 0-day Poor PEG-fused and 0-day NC rats starting at 5 weeks PO and scored –48 ± 6.8 at 8 weeks PO (**Figure 3B**). In contrast, 0-day Poor PEG-fused rats did not exhibit statistically different SFI scores compared to 0-day NC rats at any week PO. Similarly, 1-day Successful PEG-fused rats exhibited significantly higher (*P* < 0.05 or better) SFI scores of –61 ± 5.2 than 1-day Poor PEG-fused and 0-day NC rats at 8 weeks PO (**Figure 3C**). The 1-day Poor PEG-fused rats did not exhibit statistically different SFI scores compared to 0-day NC rats. These data show that VPNAs stored for 1 day and then PEG-fused enable behavioral recovery in the SD-d/SD-h sciatic nerve model.

For SD-d/Lewis-h rats, 0-day PEG-fused and 0-day NC rats exhibited similar SFI scores between 1–4 weeks PO (**Figure 3D**). 0-day PEG-fused rats had higher SFI scores than 0-day NC rats at 5 weeks PO (*P* = 0.08), but the difference was only significant (*P* < 0.05) starting at 6 weeks PO. At 8 weeks PO, 0-day PEG-fused and 0-day NC rats scored –77 ± 7.3 and –108 ± 6.6, respectively. After dividing all 0-day PEG-fused rats into 0-day Successful PEG-fused and Poor PEG-fused groups, 0-day Successful PEG-fused rats exhibited significantly better (*P* < 0.01 or better) SFI scores than 0-day Poor PEG-fused and 0-day NC rats starting at 6 weeks PO and scored –62 ± 6.3 at 8 weeks PO (**Figure 3E**). Similar SFI results were obtained in all subsequent PEG-fused groups using VPNAs stored for longer times, with Successful PEG-fused rats consistently outperforming both Poor PEG-fused and NC rats which exhibited no statistical difference at any time points (**Figure 3F–I**). 1-day Successful PEG-fused rats obtained significantly better (*P* < 0.01 or better) SFI scores than other groups starting at 6 weeks PO and scored –61 ± 3.9 at 8 weeks PO (**Figure 3F**). 2-day Successful PEG-fused rats obtained significantly better (*P* < 0.05 or better) SFI scores than other groups starting at 5 weeks PO and scored –60 ± 4.9 at 8 weeks PO (**Figure** **3G**). 3-day Successful PEG-fused rats obtained significantly better (*P* < 0.05) SFI scores than other groups at 8 weeks PO with scores of –74 ± 1.6 (**Figure 3H**). 5-day Successful PEG-fused rats obtained significantly better SFI scores than other groups starting at 6 weeks PO and scored –69 ± 3.1 at 8 weeks PO (**Figure 3I**). These data show that VPNAs stored for up to 5 days and then PEG-fused enable behavioral recovery in the SD-d/Lewis-h sciatic nerve model.

We compared successful SFI recovery rates at 8 weeks PO in all PEG-fused groups. The highest rates were observed in 0-day PEG-fused groups (**Figure 3B** and **E**; 26/40 (65%) in SD-d/SD-h rats; 9/14 (64%) in SD-d/Lewis-h rats) and 1-day PEG-fused group in SD-d/Lewis-h rats (**Figure 3F**; 10/13 (77%)). The remaining PEG-fused groups had approximately 50% success rates. No significant difference (*P* > 0.05) was found in recovery rates across PEG-fused groups using VPNAs stored for different times (**Figure 3E–I**). No significant strain difference (*P* > 0.05) was found in recovery rates across PEG-fused groups using 0-day and 1-day VPNAs (**Figure 3B** *vs.* **3E**; **Figure 3C** *vs.* **3F**).

The extent and timing of SFI recovery were compared amongst Successful PEG-fused groups across strains. Between 0-day Successful PEG-fused groups (**Figure 3B** and **E**), SD-d/SD-h rats only transiently achieved significantly (*P* < 0.05) better SFI scores than SD-d/Lewis-h rats at 6 weeks PO but not significantly better overall (*P* > 0.05). Likewise, no significant difference (*P* > 0.05) was found between 1-day Successful PEG-fused groups of both strains (**Figure 3C** and **F**). Across 0-to-5-day Successful PEG-fused groups in SD-d/Lewis-h rats (**Figure 3E-I**), 0-day and 1-day Successful PEG-fused groups transiently achieved significantly (*P* < 0.05) better SFI scores than 3-day Successful PEG-fused group at 7 weeks PO but were not significantly better overall (*P* > 0.05). Therefore, no significant difference was found in the extent or timing of locomotor recovery across Successful PEG-fused groups using VPNAs stored for different times.

### Electrophysiological assessments

**Figure 4:**
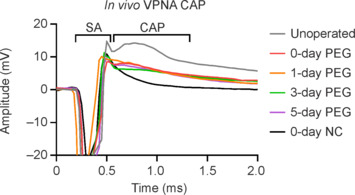
Confirmation of *in vivo* electrophysiological continuity across PEG-fused VPNAs. Representative *in vivo* compound action potential (CAP) recordings from isolated unoperated sciatic nerve, PEG-fused and NC 0-day (fresh, no storage) VPNAs, and PEG-fused 1- to 5-day VPNAs immediately following repairs. Stimulating and recording electrodes were placed on the host proximal and distal nerve segments across inserted grafts. NC: Negative control; PEG: polyethylene glycol; SA: stimulus artifact; VPNA: viable peripheral nerve allograft.

Proximal-to-distal *in vivo* CAP conduction across intact, unoperated sciatic nerves was confirmed prior to generating SL-PNIs (**Figure 4**). CAP conduction did not occur across SL-PNIs using non-fused NC VPNAs that could conduct *ex vivo* CAPs. CAP conduction was restored across SL-PNIs only after VPNAs were PEG-fused. Both PEG-fused SD-d/SD-h and SD-d/Lewis-h rats showed comparable *in vivo* CAPs. Interestingly, the amplitudes of *in vivo* CAPs across 0-day to 5-day PEG-fused VPNAs were generally similar despite their amplitude differences in *ex vivo* CAPs before transplantation (**Figure 1A**). However, many factors, such as the distance between the electrodes and/or the length and diameter of VPNAs, affected the amplitudes across recording sessions. Therefore, *in vivo* CAP recordings were assessed qualitatively, and we only reported the trend. At the end of 8 weeks PO, all rats (PEG and NC) displayed similar *in vivo* CAPs due to sufficient axonal regeneration across the lesion site (data not shown). These data support that PEG-fusion successfully fused closely apposed, open, viable host and donor axons across different VPNAs to a similar extent.

### Morphological assessments

**Figure 5:**
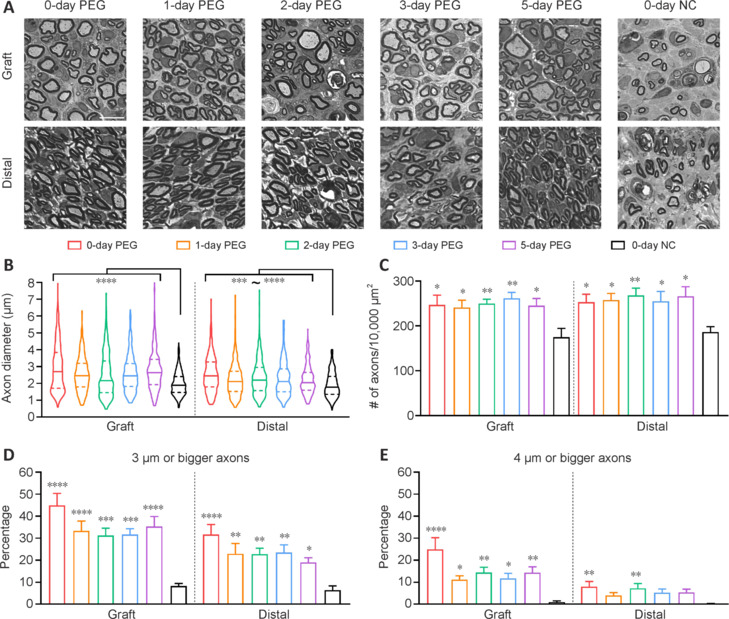
PEG-fusion repair successfully rescues many large-diameter axons in transplanted stored viable peripheral nerve allografts and distal regions. (A) Representative graft and distal cross-sections (0.5 µm) collected 8 weeks post-operatively in SD-d/Lewis-h rats across successful PEG and NC groups. Group names describe storage time plus repair method. Scale bar: 10 µm. (B–E) Morphological analyses of graft and distal cross-sections across treatment groups. One-way analysis of variance. (B) Violin plot of axon diameters. Graft regions: *F*_(5,1994)_ = 26, *P* < 0.0001; distal regions: *F*_(5,2014)_ = 16, *P* < 0.0001. *n* = 150–200 axons from at least three ROIs per sample in two samples per treatment group. (C) Average axon density. Graft regions: *F*_(5,54)_ = 3.5, *P* = 0.0076; distal regions: *F*_(5,54)_ = 3.0, *P* = 0.018. *n* = 300–550 axons from five ROIs per sample in two samples per treatment group. (D, E) Percentages of axons with ≥ 3 μm caliber or ≥ 4 μm caliber, respectively. For D, graft regions: *F*_(5,45)_ = 11, *P* < 0.0001; distal regions, *F*_(5,53)_ = 7.4, *P* < 0.0001. For E, graft regions: *F*_(5,47)_ = 9.0, *P* < 0.0001; distal regions: *F*_(5,53)_ = 3.6, *P* = 0.0071. Values are mean ± SEM. *n* of at least five ROIs per sample (quantifying at least 250 axons in total) per sample in 2 samples per treatment group. *Above each PEG-fused group indicates *P* values against the 0-day NC group. Tukey’s multiple comparison test. *P < 0.05, **P < 0.01, ***P < 0.001, ****P < 0.0001. NC: Negative control; PEG: polyethylene glycol; ROI: region of interest.

Morphometry was conducted on osmium-tetroxide-stained thick sections of the donor VPNA and host distal segments of sciatic nerves in successfully PEG-fused and NC SD-d/Lewis-h rats at 8 weeks PO (**Figure 5A**). Consistent with prior literature (Mikesh et al., 2018; Smith et al., 2025; Zhou et al., 2025), NC nerves contained much debris and mostly regenerating axons characterized by their thin myelin and small diameter (< 3 µm). In contrast, PEG-fused nerves contained less debris and interstitial space. Many large-diameter (≥ 3 µm) axons, presumed to be axons rescued from WD by PEG-fusion, were present in all PEG-fused groups (**Figure 5B**). Large-diameter axons are very infrequent within non-fused NC donor graft and host distal segments (Mikesh et al., 2018). All Successful PEG-fused groups had significantly higher (*P* < 0.01 or better) average axon diameters compared to 0-day NC group (graft: 1.99 ± 0.04 µm; distal: 1.91 ± 0.04 µm) in both the donor graft and host distal segments (**Table 2**). 0-day Successful PEG-fused group had the largest average axon diameters (graft: 2.93 ± 0.08 µm; distal: 2.55 ± 0.06 µm). Pairwise comparisons across Successful PEG-fused groups were performed (**Table 3**).

**Table 2:** Description statistics of axon morphologies

	Graft region							Distal region					
		
	0-day PEG	1-day PEG	2-day PEG	3-day PEG	5-day PEG	0-day NC		0-day PEG	1-day PEG	2-day PEG	3-day PEG	5-day PEG	0-day NC
Axon diameter (µm)	2.93±0.08	2.56±0.05	2.50±0.07	2.61±0.06	2.78±0.07	1.99±0.04		2.55±0.06	2.21±0.05	2.35±0.06	2.30±0.05	2.22±0.04	1.91±0.04
Axon density (#/10,000 µm^2^)	247±22	241±16	250±10	262±13	245±16	175±20		253±18	258±15	268±16	255±22	266±21	186±12
% of ≥ 3 µm axons	45±5.4	33±4.4	31±3.3	32±2.6	35±4.6	8.2±1.2		32±4.5	23±4.7	23±2.7	23±3.5	19±2.2	6.5±1.9
% of ≥ 4 µm axons	25±5.3	11±1.8	14±2.4	12±2.3	14±2.7	0.99±0.52		7.9±2.4	4.1±1.2	7.3±2.1	5.3±1.7	5.4±1.4	0.18±0.18

Values are mean ± SEM. NC: Negative control; PEG: polyethylene glycol.

**Table 3:** Pair-wise statistical comparison of axon diameters in graft and distal regions following viable peripheral nerve allograft repair of segmental-loss sciatic nerve injury

Graft region								Distal region						
		
	0-day PEG	1-day PEG	2-day PEG	3-day PEG	5-day PEG	0-day NC			0-day PEG	1-day PEG	2-day PEG	3-day PEG	5-day PEG	0-day NC
0-day PEG	–	***	****	**	ns	****		0-day PEG	–	****	ns	**	****	****
1-day PEG	–	–	ns	ns	ns	****		1-day PEG	–	–	ns	ns	ns	***
2-day PEG	–	–	–	ns	*	****		2-day PEG	–	–	–	ns	ns	****
3-day PEG	–	–	–	–	ns	****		3-day PEG	–	–	–	–	ns	****
5-day PEG	–	–	–	–	–	****		5-day PEG	–	–	–	–	–	***
0-day NC	–	–	–	–	–	–		0-day NC	–	–	–	–	–	–

“–” means not available. One-way analysis of variance followed by Tukey’s multiple comparison test. *P < 0.05, **P < 0.01, ***P < 0.001, ****P < 0.0001. NC: Negative control; ns: not significant; PEG: polyethylene glycol.

Axon density was also assessed across all treatment groups (**Figure 5C**). All Successful PEG-fused groups had significantly (*P* < 0.05 to *P* < 0.01) higher axon density than 0-day NC group (graft: 175 ± 20/10,000 µm^2^; distal: 186 ± 12/10,000 µm^2^) in both the donor graft and host distal regions, with densities for Successful PEG-fused groups ranging from 241 ± 16 to 268 ± 16/10,000 µm^2^ (**Table 2**). No significant difference in axon density was found across different Successful PEG-fused groups, indicating the total number of large-diameter PEG-fused axons and small-diameter regenerating axons was similar regardless of VPNA storage time.

To further explore the influence of PEG-fusion on axonal morphology, we examined the percentage of large-diameter axons (≥ 3 µm) across all treatment groups (**Figure 5D**). 0-day NC group exhibited 8.2% ± 1.2% in the donor graft and 6.5% ± 1.9% in the host distal segment. These percentages were significantly lower than those in any of the Successful PEG-fused groups (graft: *P* < 0.001 or better; distal: *P* < 0.05 or better; **Table 2**). Across Successful PEG-fused groups, 0-day Successful PEG-fused group exhibited the highest percentages of large-diameter axons (graft: 45% ± 5.4%; distal: 32% ± 4.5%), while other Successful PEG-fused groups using VPNAs stored for 1–5 days had comparable percentages in the donor graft (31%–35%) and host distal (19%–23%) portions. No statistical difference in the percentage of large-diameter axons was found across Successful PEG-fused groups, supporting that similar numbers of viable axons in stored VPNAs were rescued following PEG-fusion regardless of VPNA storage time.

Because there were still some axons in the 0-day NC group that were larger than 3 µm in both the donor graft and host distal segments, the percentage of axons with diameters ≥ 4 µm was assessed (**Figure 5E**). As shown in **Table 2**, the 0-day NC group had very low percentages of large-diameter axons (graft: 1.0% ± 0.5%; distal: 0.18% ± 0.17%). All Successful PEG-fused groups had significantly (*P* < 0.05 or better) higher percentages of large-diameter axons than the 0-day NC group in the donor graft region, with 0-day Successful PEG-fused group exhibiting the highest percentage at 25% ± 5.3% and the other Successful PEG-fused groups at 11%–14%. All randomly sampled ROIs in the donor graft segment of all Successful PEG-fused groups contained axons of ≥ 4 µm diameter; the host distal segments varied widely, with some ROIs containing none and others having up to 28% of ≥ 4 µm axons. Due to this variability, only some Successful PEG-fused groups showed significantly (*P* < 0.05 or better) higher percentages of large-diameter axons than 0-day NC group in the host distal region. Nevertheless, all Successful PEG-fused groups showed higher percentages of axons ≥ 4 µm in the host distal segment (4%–9%) compared to the 0-day NC group that had ≤ 1%, if any. This further supported that large-diameter axons observed almost exclusively in successful PEG-fused rats were viable axons in stored VPNAs that underwent successful fusion based on SFI recovery.

## Discussion

### Summary of results

The present study confirmed axonal viability in all VPNAs using both *ex vivo* CAP recordings and morphological assessments according to a storage protocol established in a previous study. This protocol preserved axonal viability for up to 9 days *ex vivo* after placing VPNAs in our storage solution that was changed daily (Zhou et al., 2023). However, we limited the storage time of VPNAs in this study to 5 days prior to their transplantation because VPNAs stored for longer periods frequently failed to conduct *ex vivo* CAPs or had smaller *ex vivo* CAP amplitudes. This decline from 5 to 9 days in storage makes longer-term storage durations less relevant for any future development of VPNA tissue banks as a viable clinical option for SL-PNI repairs.

Following *in vivo* repairs with VPNAs stored for up to 5 days, many PEG-fused rats exhibited significantly faster and better locomotor recovery, as assessed by SFI, compared to NC rats that showed no recovery over 8 weeks PO. Longer VPNA storage times did not significantly affect the rate or time course of successful SFI recovery after PEG-fusion. These results did not differ across strains.

*In vivo* CAP conduction across stored VPNAs was immediately re-established after PEG-fusion repairs but not NC repairs, consistent with prior literature (Mikesh et al., 2018; Smith et al., 2025). By 8 weeks PO, rats in all treatment groups including NCs, exhibited *in vivo* CAPs (data not shown). This result was expected due to axon regeneration by outgrowth, which was observed in nerve cross-sections in all treatment groups at 8 weeks PO. Regenerating axons are typically insufficient to produce adequate behavioral recovery after prolonged denervation of distal targets (Sakuma et al., 2016; Mikesh et al., 2018; Allgood et al., 2022), as confirmed in NC groups of both strains. In contrast, successfully PEG-fused fresh VPNA axons reinnervate many denervated distal targets that contribute to faster and better behavioral recovery (Mikesh et al., 2018), which likely would also occur in rats repaired with PEG-fused stored VPNAs.

All SD-d/Lewis-h rats classified as having successful PEG-fusion at 0 to 5 days PO had larger average axon diameters and higher axon densities in both the donor VPNA and host distal segments compared to rats classified as NC that did not receive the PEG-fusion protocol. This result is consistent with previous reports for SD-d/SD-h rats using fresh VPNAs (Mikesh et al., 2018). These differences between PEG-fused and NC repairs might be explained by a population of large-diameter axons only observed following PEG-fusion and not in NCs. These large-diameter axons were presumably rescued from WD. Longer VPNA storage times did not significantly alter average axon diameter, density, or the percentage of PEG-fused axons. We recognize that Poor PEG-fused rats were not analyzed in this study. Future studies comparing Successful and Poor PEG-fusion groups could provide insights into the morphological correlates of functional recovery.

In conclusion, this study confirms that VPNAs with viable axons enable successful *in vivo* repair of SL-PNIs via PEG-fusion using two rat allograft transplantation models. Our results achieve more satisfactory outcomes than previous attempts using stored, non-viable allografts combined with neurorrhaphy-only repairs (Evans et al., 1998, 1999).

### Possible axon populations that remain viable *ex vivo* and can be polyethylene glycol-fused *in vivo*

Longer VPNA storage times resulted in reduced *ex vivo* CAP amplitudes, indicating a progressive decline in the number of viable axons capable of conducting action potentials in VPNAs. This trend correlated with changes in *ex vivo* axonal morphology, where the percentage of well-organized axons decreased drastically and percentages of axons with abnormal myelin and/or axoplasm and disintegrated axons increased with longer VPNA storage times. However, PEG-fused VPNAs stored for 0 to 5 days had similar *in vivo* CAP peak amplitudes immediately following repairs despite amplitude differences in *ex vivo* CAPs. Additionally, PEG-fused VPNAs stored for 0 to 5 days had similar proportions of large-diameter axons in both the donor graft and host distal segments, suggesting that comparable numbers of viable axons were present and successfully PEG-fused across these VPNA storage times. These results strongly suggest that, in addition to well-organized axons, other axon populations may also remain viable and are capable of PEG-fusion. Among the other axon morphological categories, axons with abnormal myelin may still be viable because their *ex vivo* conduction may still occur, albeit diminished or blocked, while maintaining an intact axoplasm and axolemma (Smith and Hall, 1980; Uncini et al., 2024). Following successful PEG-fusion, these axons with abnormal myelin may be appropriately remyelinated by host or donor Schwann cells. In contrast, axons exhibiting abnormal axoplasm are unlikely to be viable prior to transplantation due to structural damage in the axoplasm and axolemma that precludes conduction regardless of the condition of their myelin sheaths. Disintegrated axons may represent a later stage of degeneration that starts at 5 days of storage. Therefore, it is likely that WD, though delayed by our storage protocol, eventually occurs in donor axons (Gaudet et al., 2011), at which point these axons cannot be successfully PEG-fused.

### Alternative approach

PEG-fusion repair requires closely apposed, open, and viable host and donor axons to restore both morphological and electrophysiological continuity. While our current VPNA storage protocol maintains sufficient donor axon viability for successful PEG-fusion repair up to 5 days post-storage, it does not address WD in the host distal nerve that typically initiates within 3 days post-injury. Addressing this limitation will be a key focus of our future research to extend the clinical time window for successful PEG-fusion repair.

Recent advances in tissue-engineered neuromuscular interfaces (TE-NMIs) offer an alternative strategy to address this challenge (Burrell et al., 2022). TE-NMIs consist of viable motor and sensory neurons derived from the embryonic spinal cord and dorsal root ganglia. TE-NMIs preserved the regenerative capacity of distal Schwann cells and maintained distal motor target innervation for 20 weeks PO in a porcine model of peroneal transection PNIs. This allowed successful delayed cross-suture PEG-fusion repair between the intact tibial nerve and the previously injured peroneal nerve at 20 weeks PO. The repair immediately restored electrophysiological continuity across the nerve and resulted in significantly improved electrophysiological recovery by 4 weeks PO. However, given the differences in timing, methodology, and outcome measures between that study and ours, further research is needed to make meaningful comparisons and explore how these approaches might complement each other.

### Limitations

Our protocol successfully preserves axonal viability and CAP conduction in rat VPNAs for 5 days *ex vivo*, enabling successful PEG-fusion repairs *in vivo* using stored rat VPNAs. However, this approach may not directly apply to VPNAs from other species, which may differ in nerve composition, metabolic rates, or responses to hypothermic storage (Petrov et al., 2022), potentially impacting the efficacy of the calcium-free, hypotonic storage conditions. Future studies should investigate whether this protocol can preserve VPNAs harvested from other species, particularly larger mammals commonly used in translational research, such as rabbits, pigs, or non-human primates. This would be a critical step in validating the generalizability of this storage protocol to human VPNAs.

This study uses SFI as the primary behavioral outcome, which is predominantly motoneuron-driven. Future studies should provide a more comprehensive evaluation of both motor and sensory recovery by including other metrics such as the Von Frey filament test.

## Conclusion

This study demonstrates that both *ex vivo* and *in vivo* CAPs may be used to quality-control rat sciatic VPNAs for their suitability for PEG-fusion of SL-PNIs. VPNAs maintained for up to 5 days using our *ex vivo* storage protocol can effectively restore locomotor behaviors following PEG-fusion repairs of SL-PNIs. Importantly, many axons in VPNAs stored using this protocol did not undergo rapid WD *ex vivo* and remained viable, a condition that is critical for successful PEG-fusion. Therefore, this study shows PEG-fusion can be achieved without freshly obtained VPNAs, demonstrating its clinical applicability beyond acute scenarios. Beyond its application for rat VPNA storage, our current protocol may also be valuable for preserving grafts of larger species or humans. For example, autografts undergoing investigation in a PEG-fusion clinical trial (Consortium, 2024) could benefit from this storage protocol. Further research should test other conditions that may affect and improve axon viability to maximize the storage time of VPNAs that can still be successfully PEG-fused. Establishing a VPNA tissue bank could further enhance the clinical feasibility and scheduling flexibility of PEG-fusion repairs, offering better clinical outcomes following SL-PNIs than the current standard interventions.

## Data Availability

*No additional data are available*.
